# A Comparison of Singlet Oxygen Explicit Dosimetry (SOED) and Singlet Oxygen Luminescence Dosimetry (SOLD) for Photofrin-Mediated Photodynamic Therapy

**DOI:** 10.3390/cancers8120109

**Published:** 2016-12-06

**Authors:** Michele M. Kim, Rozhin Penjweini, Nathan R. Gemmell, Israel Veilleux, Aongus McCarthy, Gerald S. Buller, Robert H. Hadfield, Brian C. Wilson, Timothy C. Zhu

**Affiliations:** 1 Department of Radiation Oncology, University of Pennsylvania, Philadelphia, PA 19104, USA; mickim@sas.upenn.edu (M.M.K.); Rozhin.Penjweini@uphs.upenn.edu (R.P.); 2 Department of Physics and Astronomy, University of Pennsylvania, Philadelphia, PA 19104, USA; 3 Department of Electronic and Nanoscale Engineering, University of Glasgow, Glasgow G12 8LT, UK; Nathan.Gemmell@glasgow.ac.uk (N.R.G.); Robert.Hadfield@glasgow.ac.uk (R.H.H.); 4 Princess Margaret Cancer Centre, University of Toronto, ON M5G 1L7, Canada; israel.veilleux@uhnresearch.ca (I.V.); wilson@uhnresearch.ca (B.C.W.); 5 Institute of Photonics and Quantum Sciences, Heriot-Watt University, Edinburgh EH14 4AS, UK; A.McCarthy@hw.ac.uk (A.M.); g.s.buller@hw.ac.uk (G.S.B.)

**Keywords:** photodynamic therapy, singlet oxygen explicit dosimetry (SOED), singlet oxygen luminescence dosimetry (SOLD), Photofrin, oxygen

## Abstract

Accurate photodynamic therapy (PDT) dosimetry is critical for the use of PDT in the treatment of malignant and nonmalignant localized diseases. A singlet oxygen explicit dosimetry (SOED) model has been developed for in vivo purposes. It involves the measurement of the key components in PDT—light fluence (rate), photosensitizer concentration, and ground-state oxygen concentration ([^3^*O*_2_])—to calculate the amount of reacted singlet oxygen ([^1^*O*_2_]_rx_), the main cytotoxic component in type II PDT. Experiments were performed in phantoms with the photosensitizer Photofrin and in solution using phosphorescence-based singlet oxygen luminescence dosimetry (SOLD) to validate the SOED model. Oxygen concentration and photosensitizer photobleaching versus time were measured during PDT, along with direct SOLD measurements of singlet oxygen and triplet state lifetime (*τ*_Δ_ and *τ_t_*), for various photosensitizer concentrations to determine necessary photophysical parameters. SOLD-determined cumulative [^1^*O*_2_]_rx_ was compared to SOED-calculated [^1^*O*_2_]_rx_ for various photosensitizer concentrations to show a clear correlation between the two methods. This illustrates that explicit dosimetry can be used when phosphorescence-based dosimetry is not feasible. Using SOED modeling, we have also shown evidence that SOLD-measured [^1^*O*_2_]_rx_ using a 523 nm pulsed laser can be used to correlate to singlet oxygen generated by a 630 nm laser during a clinical malignant pleural mesothelioma (MPM) PDT protocol by using a conversion formula.

## 1. Introduction

Photodynamic therapy (PDT) is an evolving treatment modality for many cancers such as microinvasive lung cancer, obstructing lung cancer, and obstructing esophageal cancer, for premalignant diseases such as actinic keratosis, and non-oncologic conditions such as age-related macular degeneration [1,2,3]. PDT is advantageous for its low systemic toxicity, lack of induced resistance, repeatability, and preservation of normal tissue structure. Widespread use of PDT has been restricted by the difficulty in accurately quantifying the effective treatment dose. PDT is dynamic and multifaceted, with complex and dynamic interactions between treatment light at a particular wavelength, photosensitizer (PS), and tissue oxygen ([^3^*O*_2_]) [3]. In a typical photodynamic process, the photosensitizer is excited by the treatment light and enters the excited singlet state. This singlet state undergoes intersystem crossing to the triplet state. This triplet state can react directly with molecular substrates or transfer a hydrogen atom or an electron to ^3^O_2_ to produce radicals or radical ions in a type I process [4]. Most clinically relevant photosensitizers undergo type II processes in which the triplet state transfers energy to ground-state oxygen, ^3^O_2_, to produce singlet oxygen, ^1^O_2_ [4], which is the main photocytotoxic agent leading to cell death and therapeutic response [5,6].

PDT dosimetry has so far involved the prescription of an administered drug dose and a light fluence (energy per unit area). This is often inadequate due to the variation in photosensitizer localization from patient to patient as well as within the tumor environment [7,8]. Furthermore, tissue and blood oxygenation are key components for PDT and will affect not only the production of ^1^O_2_ but also the tissue optical properties [9,10]. In turn, the penetration of light is dependent on the tissue optical properties [11]. Simplistically, PDT dose may be defined as the time integral of the photosensitizer concentration and the light fluence. However, this does not take into account the effect of tissue hypoxia. In a hypoxic environment, the production of ^1^O_2_ will be lower than expected and treatment outcome will not be predictable [7,8].

For these compelling reasons, the use of singlet oxygen concentration, [^1^*O*_2_] as the dosimetric measure has been suggested. Direct measurement of ^1^O_2_ by its near-infrared luminescence emission is technically challenging in vivo due to the weakness of the signal and the short lifetime of ^1^O_2_, ~30–180 ns [12,13]. Hence, a macroscopic singlet oxygen explicit dosimetry (SOED) model has been previously developed and studied for various sensitizers in vivo [8,14,15,16]. In the present work, SOED was compared in solutions to direct singlet oxygen luminescence dosimetry (SOLD) [17,18]. The relevant photophysical parameters for the macroscopic model were verified by performing explicit dosimetry of oxygen concentration and photosensitizer concentration. In performing a direct comparison between SOED- and SOLD-measured ^1^O_2_, the use of SOED in scenarios where direct luminescence detection is difficult is validated. Furthermore, an analysis is provided to show that SOLD measured using a 523 nm pulsed laser (currently required by the availability of lasers with suitable pulse length, repetition frequency, and energy) is well-correlated to singlet oxygen generated by Photofrin by a CW 630 nm laser during PDT, by correcting for the tissue optical properties at the two wavelengths.

## 2. Materials and Methods

### 2.1. SOED Model In Vitro and In Vivo

Singlet oxygen produced during illumination was calculated using an explicit dosimetry model. Based on type II processes modeled, a set of coupled differential equations has been established for the photophysical reactions [8,16,19].

A set of simplified differential equations that are valid over time scales of a few seconds to hours can be used to describe the interactions of singlet oxygen concentration, [^1^*O*_2_], photosensitizer concentration, [*S*_0_], the ground-state oxygen concentration, [^3^*O*_2_], and the reacted singlet oxygen concentration, [^1^*O*_2_]_rx_ for the in vitro scenario with parameters (and *k*_i_) defined in Table 1 (and its footnote) [19]:

(1) [O21]=ξτΔ[O23][O23]+βϕ[S0]1(σ([S0]+δ)+1)

(2) d[S0]dt=−ξ[O23][O23]+βϕ[S0](σ([S0]+δ)(σ([S0]+δ)+1))

(3) d[O23]dt=−ξ[O23][O23]+βϕ[S0](σ([S0]+δ)+k7[A]τΔ(σ([S0]+δ)+1))

(4) [O21]rx=∫ξ[O23][O23]+βϕ[S0]1(σ([S0]+δ)+1)dt

where *σ = k*_1_/(*k*_6_ + *k*_7_[*A*]), *ξ = Φ*_Δ_(*ε*/*hν*), *τ*_Δ_^−1^ *= k*_6_ *+ k*_7_[*A*], and *β* = *k*_4_/*k*_2_. Here, *Φ*_Δ_ is the singlet oxygen quantum yield in the aqueous Intralipid medium. The parameters used for the calculation for each phantom are summarized in Table 1. For studies without NaN_3_, the ^1^O_2_ quencher used in solutions, [*A*] = 0. For the in vivo scenario, Equations (2) and (3) can be rewritten as [8,14,15,16,20]:

(5) d[S0]dt=−ξ[O23][O23]+βϕ[S0]σ([S0]+δ)

(6) d[O23]dt=−(ξϕ[S0][O23]+β)[O23]+g(1−[O23][O23](t=0))

where we assumed that *σ*([*S*_0_] *+ δ*) << 1. Here we have added an oxygen perfusion term to account for vasculature in vivo, and the value of *g* = 0.7 μM/s for Photofrin.

Equation (5) can be rewritten as the following:

(7) −d[S0]dt/ϕ[S0][O23][O23]+β=ξσ([S0]+δ)

The left-hand side of Equation (7) versus [*S*_0_](*t*) gives the values of *δ* and *σ* used in Table 1. The photobleaching rate (*−d*[*S*_0_]/*dt*) was determined at each time point, along with the values of *φ*, [^3^*O*_2_], [*S*_0_], and *β* for the calculation of the left-hand side of Equation (7). A linear fit to the data yields a value for the intercept and slope, and the intercept divided by the slope gives *δ* and the slope divided by *ξ* gives *σ*. A recent review lists known values for in vivo photophysical parameters for many photosensitizers used clinically [19]. The SOED-calculated solutions were compared with the oxygen and [*S*_0_](*t*) concentrations. All calculations were performed using Matlab 2014b (MathWorks, Natick, MA, USA).

### 2.2. SOLD Instrumentation

Singlet oxygen luminescence dosimetry (SOLD) was performed using a compact, fiberoptic near-infrared probe-based system [21,22]. The probe was coupled to a compact InGaAs/InP single photon avalanche diode (SPAD) detector (Micro Photon Devices, Bolzano Italy). Samples were illuminated with a 523 nm wavelength pulsed laser (QL-523-200-S, CrystaLaser, Reno, NV, USA) coupled into the delivery fiber via a collimation package. Patterned time gating was used to limit the unwanted dark counts and eliminate the strong photosensitizer luminescence background. The luminescence signal from singlet oxygen at 1270 nm was confirmed through spectral filtering and lifetime fitting for Photofrin.

**Figure 1:**
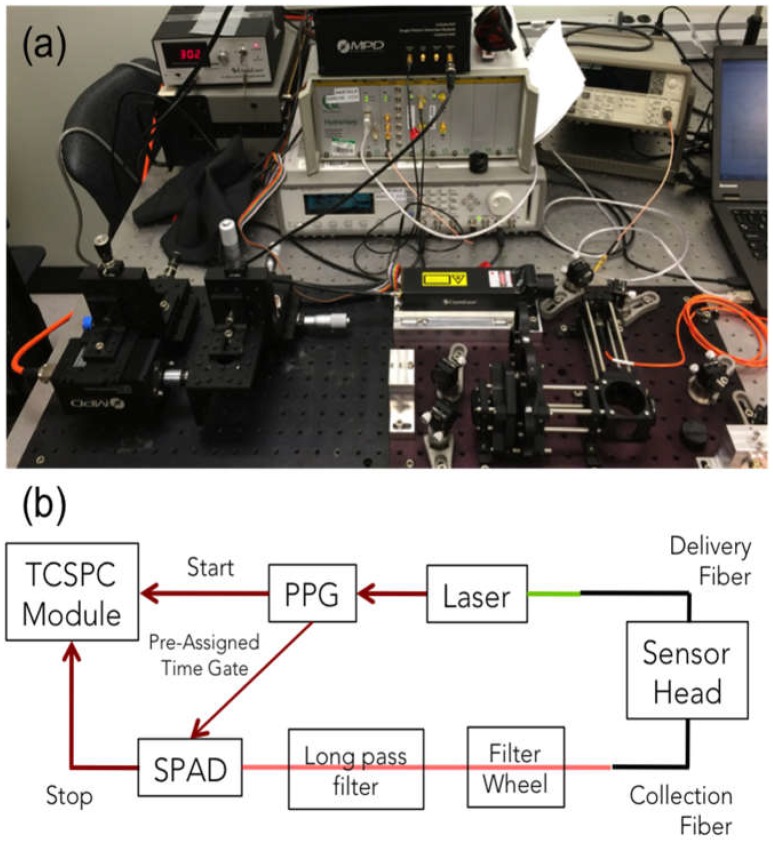
Singlet oxygen luminescence dosimetry (SOLD) instrumentation setup (**a**) on an optical bench; and (**b**) schematic diagram of the experimental arrangement. PPG—pulse pattern generator; SPAD—single photon avalanche diode; TCSPC—time-correlated single-photon counting.

Figure 1 shows a photograph and schematic of the experimental setup. The pulsed laser was coupled into the delivery fiber. The laser also outputs an electrical signal that is sent to a programmable pulse pattern generator (PPG) (Agilent 81110A, Keysight Technologies, Inc., Santa Rosa, CA, USA). Each pulse generates outputs on two separate channels, each with pulse shape designed to match the intended input. The first output is a single pulse sent to the “start” channel of the time-correlated single-photon counting (TCSPC) module (HydraHarp, PicoQuant GmbH, Berlin, Germany), while the second is a pattern of pulses sent to the SPAD control module. The SPAD is turned on for a preassigned time, only when the control module receives a pulse from the PPG.

The TCSPC module generates a timing histogram of photon counts versus time. The background was removed by subtracting the histogram taken through a 1210 nm filter from that through the 1270 nm filter. Equation (8) describes the [^1^*O*_2_] signal as a function of time following a short illumination pulse.

(8) [O21](t)=NσA[S0]ΦΔτΔτt−τΔ(e−t/τt−e−t/τΔ)

The cumulative SOLD singlet oxygen count can be calculated as the integral of Equation (8) per *τ_R_* [23].

(9) ∫0∞1τR[O21](t)dt=NσA[S0]ΦΔτΔτR

where *N* is the number of photons in the illumination pulse, *σ_A_* is the PS absorption cross-section (*σ_A_* = (*ε*/*N_A_*) × 10^9^), *N_A_* is Avogadro’s constant (6.022 × 10^23^), *ε* is the extinction coefficient, and *τ_R_* is the ^1^O_2_ phosphorescence lifetime (*k*_6_^−1^). A fit of the background-subtracted histograms was performed to Equation (8) (with a *y*-axis offset as a free parameter to account for any change in the background level) using Origin software with a Levenberg–Marquardt algorithm to iterate the parameter values.

### 2.3. Measurements in Tissue-Simulating Phantoms

Explicit dosimetry of phantom studies was performed using tissue-simulating liquid phantoms. A lipoprotein colloidal suspension, Intralipid (Fresenius Kabi, Uppsala, Sweden) was added to solutions to provide optical scattering. A broad beam (0.4 cm radius) was produced by a fiber with a microlens attachment (Pioneer Optics Company, Bloomfield, CT, USA) onto the side of cuvette phantoms. Oxygen measurements were made with a bare-fiber OxyLite probe (Oxford Optronix, Oxford, UK) on the side closest to beam entry at the center of the field. In the in vitro setup, there is very little oxygen diffusion at the point of measurement, so that oxygen measurements were performed with brief interruptions of the excitation laser at 1–30 s intervals. The oxygen partial pressure was measured in mmHg and converted to μM by using a factor of *α* = 1.3 [14,24]. PS concentration was determined by obtaining fluorescence spectra produced by Photofrin excited by the treatment light. Spectral analysis was performed using single value decomposition fitting of the characteristic Photofrin peak [25].

### 2.4. Comparison of 630 nm and 523 nm SOED

Optical properties from multiple sites of several different patients undergoing treatment for pleural-PDT were determined from absorption spectra using a white light source (Avantes, Little Rock, AK, USA) connected to a multifiber contact probe, as described elsewhere [26,27]. The multifiber probe has a source fiber attached to a halogen white light source. This was used to obtain broadband reflectance at multiple source-detector separations. The diffusion equation was used to determine the solution for diffuse reflectance for a semi-infinite medium with steady-state excitation. The longitudinal distribution of *φ* for different tissue optical properties was determined using an analytical formula [28] based on Monte Carlo simulations [29,30,31,32]. To obtain the corresponding temporal changes of [*S*_0_] and [^3^*O*_2_], the information of *φ* distribution, the magnitude of the Photofrin-specific reaction-rate parameters, and the measured photosensitizer concentrations [33] were passed to the time (*t*)-dependent differential Equations (5) and (6) for a given treatment time point, which were then used to calculate [^1^*O*_2_]_rx_ using Equation (4).

## 3. Results

### 3.1. SOED Photophysical Parameters

**Figure 2:**
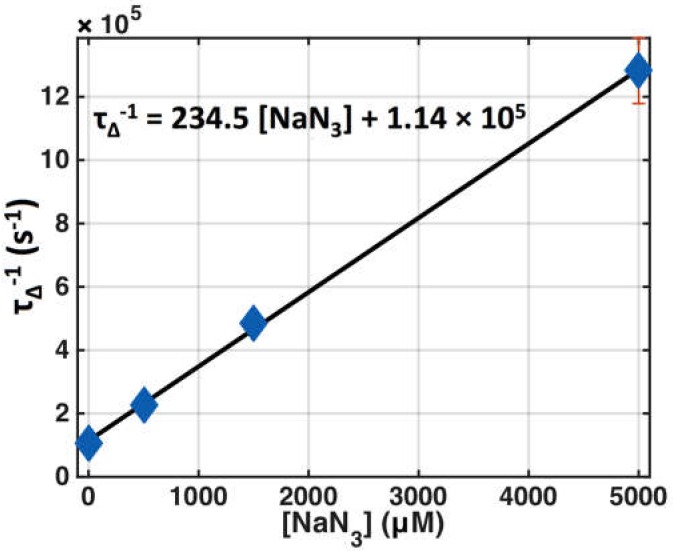
Singlet oxygen lifetime (*τ*_Δ_) changes due to quenching with various concentrations of sodium azide (NaN_3_) for Photofrin (50 μM) in MeOH, *τ*_Δ_^−1^ = *k*_6_ + *k*_7_[*A*]. Symbols represent measured data and the solid line is the best linear fit.

Photophysical parameter values for Photofrin were determined for in vitro macroscopic modeling from the literature as well as measurements to be used in the calculation of [^1^*O*_2_]_rx_. These parameters are listed in Table 1. Figure 2 shows the SOLD measurement of singlet oxygen lifetime as a function of the concentration of added sodium azide (NaN_3_), which is a potent singlet oxygen-specific quencher, for Photofrin in MeOH solution. The intercept of the linear fit (solid line in Figure 2) corresponds to *k*_6_ and the slope corresponds to the value for *k*_7_.

### 3.2. SOED in Phantom

Photofrin phantoms with Intralipid as optical scatterer and absorption due to both the photosensitizer and water (or Intralipid) were used to measure the time dependence of [^3^*O*_2_] and PS concentration, [*S*_0_], under CW 630 nm laser excitation. [^3^*O*_2_](t) was measured using an oxygen phosphorescence probe and the photophysical parameters taken from Table 1.

**Figure 3:**
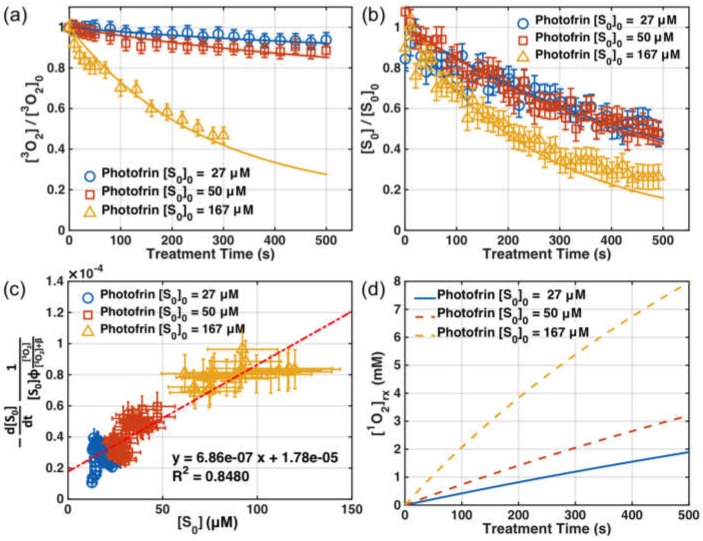
Comparison of measured and singlet oxygen explicit dosimetry (SOED)-calculated values of (**a**) [^3^*O*_2_](*t*) and (**b**) [*S*_0_](*t*) at *d* = 0 for three initial photosensitizer concentrations, [*S*_0_]_0_ = 27, 50, 167 μM. Measurements of ground-state oxygen were made at 5–30 s intervals, while photosensitizer spectra were obtained every 10 s. The average initial [^3^*O*_2_]_0_ value was 160.4 μM. (**c**) The left-hand side of Equation (7) versus the Photofrin concentration, with the line of best fit. The slope of the fit was (6.86 ± 0.6) × 10^−7^ mM·s^−1^·mW^−1^·cm^2^ and the intercept was (1.78 ± 0.25) × 10^−5^ s^−1^·mW^−1^·cm^2^, resulting in a value of *δ* = 25 ± 4.3 μM. (**d**) Calculated volume-averaged [^1^*O*_2_]_rx_ over time. The light fluence rates used in the experiment were *φ*_0_ = 45, 38, and 42 mW/cm^2^ for each sensitizer concentration. The symbols are measured values in Figure 3a,b and are calculated values using Equation (7) for Figure 3c. The lines are SOED-calculated results for Figure 3a,b,d, and the line of best fit for Figure 3c.

Figure 3a,b show the measured [^3^*O*_2_] and [*S*_0_] at just below the surface (*d* = 0) versus time in an Intralipid phantom (with *μ_s_’* = 0.2 cm^−1^) for three different initial Photofrin concentrations (27, 50, 167 mM). The symbols are measured values and the lines are SOED-calculated results. Figure 3c shows the PS photobleaching rate per PDT dose, −d[S0]dt1[S0]ϕ[O23]/([O23]+β) versus [*S*_0_]. The symbols are calculated values using Equation (7), and the line is the best linear fit. Figure 3d shows the expected SOED-calculated cumulative reacted singlet oxygen concentration, [^1^*O*_2_]_rx_, during illumination.

### 3.3. SOED/SOLD Comparison in Solution

**Figure 4:**
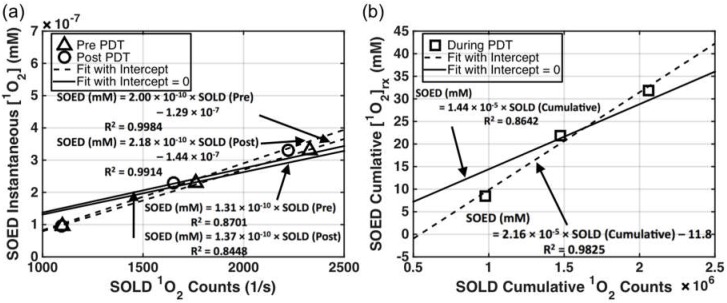
(**a**) Comparison of SOLD-obtained ^1^O_2_ counts (Equation (8)) per accumulation time (in seconds) at 523 nm and SOED-calculated instantaneous [^1^*O*_2_] (Equation (1)) for Photofrin concentrations in MeOH of 17, 50, and 83 μM, and light fluence *φ*_0_ = 30 mW/cm^2^. The initial oxygen concentration was measured as 175 ± 6 μM. (**b**) Comparison of SOLD cumulative ^1^O_2_ counts (Equation (9)) and reacted singlet oxygen concentration ([^1^*O*_2_]_rx_) calculated with SOED (Equation (4)) for Photofrin concentration of 17, 50, and 83 μM. PDT was performed with 523 nm light at *φ*_0_ = 30 mW/cm^2^ for 900 s.

The singlet oxygen generated in Photofrin-containing solutions was calculated using SOED and the results were compared to SOLD-determined luminescence counts of ^1^O_2_. The latter was correlated with the amount of ^1^O_2_ produced instantaneously and cumulatively. Instantaneous [^1^*O*_2_] accounts for the singlet oxygen generated for each pulse of laser excitation, while cumulative [^1^*O*_2_]_rx_ is the integral of all singlet oxygen produced during the entire illumination time over the entire illumination volume. The agreement between the two methods (SOED and SOLD) is shown in Figure 4: (a) shows SOLD counts per accumulation time (in seconds, *t* = 300 s before and after PDT) and (b) shows cumulative SOLD counts over the entire treatment time of 900 s. Photofrin was dissolved in MeOH solution.

## 4. Discussion

### 4.1. SOED and SOLD Intercomparison

The SOED calculated [^1^*O*_2_] value in solution in Figure 4a corresponded to the volumetric averaged instantaneous singlet oxygen concentration over a volume of 1 cm depth and 1 cm^2^ area. SOED-calculated [^1^*O*_2_]_rx_ in Figure 4b corresponded to the volumetric average reacted singlet oxygen concentration of the same 1 cm^3^ volume. In these solutions, the light fluence was calculated by introducing attenuation that was only due to the photosensitizer absorption, since no scatterer was added and solutions were of pure Photofrin: *φ = φ*_0_ *exp*(*−μ_a_ × d*), where *φ*_0_ is the light fluence rate (mW/cm^2^) measured directly in the back of the front wall of the solution facing the laser. Absorption coefficients (*μ*_a_) were 0.15, 0.45, and 0.74 cm^−1^ for Photofrin concentrations of 17, 50, and 83 mM, respectively, at 523 nm.

In order to experimentally determine the photophysical parameters of the spontaneous phosphorescence rate of ^1^O_2_ to ^3^O_2_ (*k*_6_) and the bimolecular reaction rate of ^1^O_2_ with the substrate (*k*_7_) in Photofrin phantoms, photosensitizer triplet-state and singlet oxygen lifetime measurements were obtained using the SOLD system. Varying amounts of the singlet oxygen quencher, sodium azide (NaN_3_), were added to the Photofrin–MeOH solutions. The resulting fits to obtain *k*_6_ and *k*_7_ are shown in Figure 2. For Photofrin with NaN_3_, *k*_6_ was found to be 1.14 × 10^5^ s^−1^ (the intercept of the line of best fit in Figure 2) and *k*_7_ was found to be 235 μM^−1^·s^−1^ (the slope of the line of best fit in Figure 2). *k*_7_ is pH-dependent, but is in the range of the reported value of 300–400 μM^−1^·s^−1^ for the quenching rate constant in water [38]. These values were used to calculate *τ*_∆_ for the in vitro condition (without NaN_3_) and the in vivo condition (taken from the literature for biological tissue [34]). Assuming that *k*_7_ for NaN_3_ is greater than or equal to that of in vivo conditions (assuming biological tissue is less efficient than NaN_3_ in quenching ^1^O_2_), it can be estimated that in vivo acceptor concentration [*A*] ≥ 10^7^ (s^−1^)/235 mM^−1^·s^−1^ = 42 mM. This value is much higher than [*A*] = 0.83 mM in the literature [10], but we feel that it is more reasonable since the singlet oxygen lifetime in vivo, *τ*_∆_, does not change for reacted singlet oxygen concentrations [^1^*O*_2_]_rx_ as high as 12 mM [16], indicating there are still plenty of acceptors in vivo at this level.

**Figure 5:**
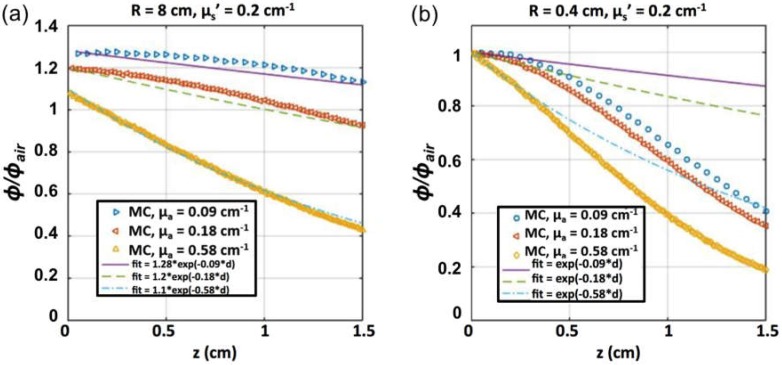
Monte Carlo (MC) simulation of fluence rate distribution by a circular beam of radius (**a**) 0.4 cm and (**b**) 8 cm incident on a semi-infinite liquid surface as a function of depth (*d*) for *μ_a_* = 0.09, 0.18, and 0.58 cm^−1^ and *μ*_s_’ = 0.2 cm^−1^. Fits of exponential forms are shown along with the MC data. The exponential form of *e^−μ_a_d^* fits the simulation well up to a depth of 0.4 cm, while overestimating *φ*/*φ*_0_ at larger depths. Broad-beam simulations agree with the simple exponential form up to a depth of 1.3 cm.

The light fluence rate distribution in a semi-infinite medium as a function of distance (*d*) was calculated by a Monte Carlo (MC) simulation [39] of a circular parallel beam (diameter 0.8 cm, Figure 5a) and broad beam (diameter 16 cm, Figure 5b) for absorption coefficient (*μ_a_*) of 0.09, 0.18, and 0.58 cm^−1^, and reduced scattering coefficient (*μ_s_’*) of 0.2 cm^−1^. The resulting *φ*/*φ*_0_ is shown in Figure 5 along with an exponential fit based on *μ_a_*. For the tissue-simulating phantoms with Photofrin shown in Figure 3, *φ*_0_ is the measured local fluence rate at the front inner surface of the phantom facing the laser and *d* is the depth from surface. At 630 nm, *μ_a_* = 0.09, 0.18, and 0.58 cm^−1^ for Photofrin concentrations of 27, 50, and 167 mM, respectively. It is clear that the function *e^−μ_a_d^*, while working well for the broad beam, does not work very well for the 0.8 cm diameter beam at the deepest depths investigated. As a result, MC-generated light fluence rate *φ*/*φ*_0_ was used directly for the SOED calculations in phantom.

SOED calculations of singlet oxygen concentration are highly dependent on the photophysical parameters used as input (Table 1). In turn, these parameters depend on the photosensitizer used, as well as the treatment environment. The necessary parameters for Photofrin-mediated PDT for in vitro studies were validated with explicit measurements of the [^3^*O*_2_] and [*S*_0_]. In particular, the consumption rate of [*S*_0_] per PDT dose was used to determine a more accurate value of *σ* (slope/*ξ*) and *δ* (intercept/slope) for the experimental setup used (Figure 3c and Equation (7)). This was used to determine *δ* and *σ* using a method from Reference [40] that is also described in Section 2.1. Photosensitizer concentration was measured over time to determine the photobleaching rate (*−d*[*S*_0_]/*dt*) and [*S*_0_]. Along with the measured [^3^*O*_2_], the PS photobleaching rate per PDT dose can be calculated and plotted as in Figure 3c. The slope and intercept of the fit to the data are used to calculate *δ* and *σ*. The value for *ξ* in vitro was calculated by the definition of *ξ* provided in Table 1. The resulting values were *δ* = 25 ± 4.3 μM and *σ* = (6.6 ± 7) × 10^−5^ μM^−1^. The value of *β* was set to be 11.9 μM for this set of experiments [34]. Figure 3a,b show the SOED calculations using Equations (2) and (3), which agree with [^3^*O*_2_](*t*) and [*S*_0_](*t*) measurements at surface (*d* = 0 cm) of the Intralipid phantom. Figure 3d shows the magnitude of SOED-calculated [^1^*O*_2_]_rx_ using Equation (4) for Photofrin to be in the mM range.

It can be concluded from the intercomparison of SOED and SOLD in Photofrin solutions (Figure 5b) that the cumulative SOLD [^1^*O*_2_] counts, [*SOLD*], and SOED-calculated [^1^*O*_2_]_rx_ values track each other very well (*R*^2^ = 0.98) for Photofrin, with a conversion factor of the following form:

(10) [O21]rx(mM)=(2.16×10−5)×[SOLD]−11.8

The ratio of slopes between the two panels ((a) and (b)) in Figure 4 is 9.6 × 10^−6^ s, which is consistent with the value of *τ*_Δ_ obtained (9.4 × 10^−6^ s). The reason for the intercept is not known, and a linear fit without intercept reduces the correlation (*R*^2^ = 0.86). The good correlation of SOED-calculated [^1^*O*_2_] and [*SOLD*] demonstrates that SOED can be utilized in scenarios where direct phosphorescence measurement of ^1^O_2_ is difficult.

### 4.2. Feasibility of Using SOLD at 523 nm for Predicting [^1^O_2_]_rx_ at 630 nm

**Figure 6:**
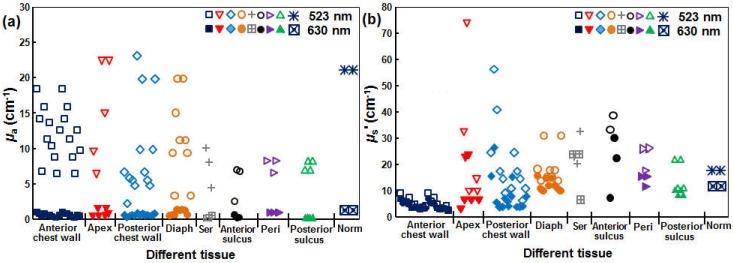
(**a**) Tissue *μ*_a_’ and (**b**) *μ*_s_’ at 523 (hollow symbols) and 630 (filled symbols) nm measured in vivo in malignant pleural mesothelioma (MPM) patients.

Currently, the only available pulsed laser suitable for the SOLD application (CrystaLaser, QL-523-200-S, CrystaLaser, Reno, NV, USA) is at 523 nm. As a result, the effective tissue-sampling depth for [^1^*O*_2_] is not the same as that of the 630 nm treatment light used clinically with Photofrin. Figure 6 shows the measured values *μ*_a_ and *μ*_s_’ in various sites measured in vivo in patients, including the anterior chest wall, apex of the heart (apex), posterior chest wall, diaphragm (diaph), serratus (ser), anterior sulcus, posterior sulcus, pericardium (peri), and normal (norm) tissue. Patients were undergoing an institutional review board (IRB)-approved pleural mesothelioma Photofrin-PDT clinical protocol at the University of Pennsylvania. These optical properties were measured using a custom-built multifiber contact probe for absorption spectroscopy [27]. The measured optical properties include *μ*_a_ and *μ*_s_’ for tissue as well as Photofrin.

**Figure 7:**
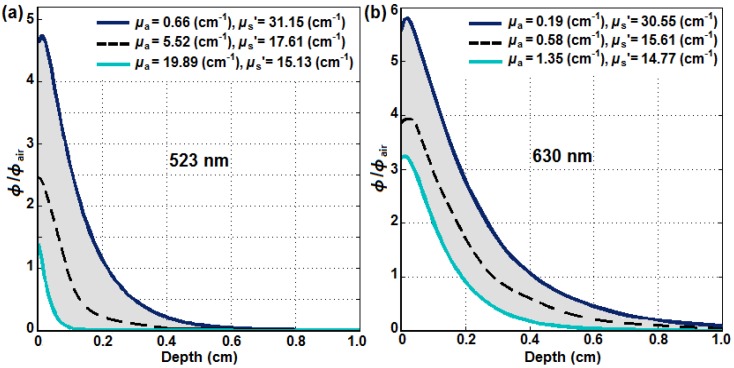
The ratio of *φ* and in-air fluence rate (*φ*_air_) versus tumor depth for (**a**) 523 nm and (**b**) 630 nm optically broad laser beams on an air–tissue interface using an analytical formula that fits MC simulation [29,30,31,32] and optical properties obtained in in vivo MPM patients (Figure 4).

Using an analytical fit [28] to MC simulations [29,30,31,32], the longitudinal distribution of *φ* in tissue with different optical properties was calculated. Figure 7 shows the ratio of *φ* and in-air fluence rate (*φ*_air_) versus tumor depth for (a) 523 nm and (b) 630 nm. The gray area shows the region of *φ*/*φ*_air_ with the upper and lower bounds of the tissue optical properties obtained in vivo as dark blue and light blue, respectively. The dashed black lines show the calculated light fluence distribution using the mean optical properties of *μ_a_* = 5.52 cm^−1^ and *μ_s_’* = 17.61 cm^−1^ for 523 nm and *μ_a_* = 0.58 cm^−1^ and *μ_s_’* = 15.61 cm^−1^ for 630 nm. As expected, the optical penetration is much deeper at 630 nm than at 523 nm in in vivo malignant pleural mesothelioma (MPM) patients.

**Figure 8:**
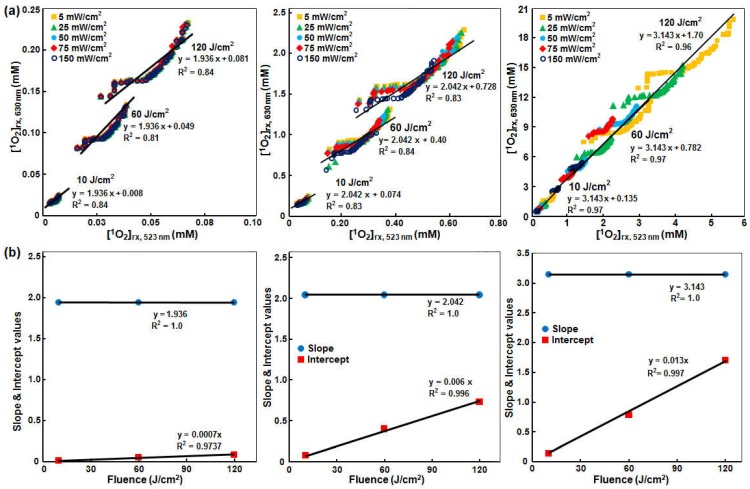
(**a**) [^1^*O*_2_]_rx_ calculated at 630 nm and 523 nm for different total fluence (*φ* = 10, 60, 120 J/cm^2^) for mean Photofrin concentrations (*c*) of (from left to right) 0.21, 2.1, and 21 μM. Absorption and scattering coefficients were obtained at the two wavelengths from Figure 6. SOED calculation of [^1^*O*_2_]_rx_ used Equations (5) and (6) and averaged over a 1 cm depth and 1 cm^3^ volume using photophysical parameters listed in Table 1 for the in vivo conditions; (**b**) Slope and intercept of the correlation of [^1^*O*_2_]_rx_ at 630 nm and 523 nm as a function of fluence and PS concentration.

The *φ* distributions were then used to calculate the reacted singlet oxygen concentration for the two wavelengths, in order to study whether SOLD signals measured at 523 nm can be used to monitor [^1^*O*_2_]_rx_ at 630 nm. MPM PDT is currently performed at 630 nm. Correlation between the calculated [^1^*O*_2_]_rx_ for 630 and 523 nm is shown in Figure 8. *μ_a_* ranges from 0.66 to 23.1 cm^−1^ at 523 nm and 0.17 to 1.35 cm^−1^ at 630 nm, while *μ_s_’* ranges from 2.80 to 73.7 cm^−1^ at 523 nm and 2.55 to 30.5 cm^−1^ at 630 nm (Figure 6). In order to investigate the effects of different *φ* on the [^1^*O*_2_]_rx_, the SOED calculations were repeated for *φ* = 5, 25, 50, 75, and 150 mW/cm^2^. Different colors of symbols represent different *φ*. The black solid lines are the best fits in Figure 8b. At 523 nm, the range of [^1^*O*_2_]_rx_ changed from 0–0.1, 0–0.63, and 0–5.6 mM for PS concentrations of 0.21, 2.1, and 21 µM, respectively, while the range of [^1^*O*_2_]_rx_ at 630 nm changed from 0–0.25, 0–2.5, and 0–20 mM, respectively, for the same PS concentrations.

The resulting correspondence for a range of PS concentrations (*c*) of 0.21, 2.1, and 20.1 mM (based on the average Photofrin concentration obtained in patients) and light fluence of 10–120 J/cm^2^ [33] can be expressed as:

(11) [O21]rx(630 nm)=a(c)[O21]rx(523 nm)+b(c,ϕ)

where

(12) a(c)=0.05814c+1.922

and

(13) b(c,ϕ)=(0.000618c−0.000033)ϕ

where *c* is the PS concentration (in μM) and *φ* is the light fluence (in J/cm^2^). We thus conclude that SOLD measurement at 532 nm can be used to monitor [^1^*O*_2_]_rx_ at 630 nm if a conversion formula (Equations (11)–(13)) is used to convert the measured SOLD signal.

When SOLD signal from patients are used to determine the generation of singlet oxygen, it is important to develop a tissue optical properties correction factor to account for the absorption and scattering of luminescence by tissue, similar to the optical properties correction factor needed for using fluorescence to determine the PS concentration [14]. This is beyond the scope of this paper.

## 5. Conclusions

Direct singlet oxygen luminescence dosimetry (SOLD) measurements were compared with singlet oxygen explicit dosimetry (SOED) calculations for phantoms using Photofrin. Oxygen and PS concentration measurements were compared with SOED predictions to validate the SOED model and to obtain the photophysical parameters (Table 1, *δ* = 25 ± 4.3 μM and *σ* = (6.6 ± 7) × 10^−5^ μM^−1^ in vitro). Using lifetime measurements obtained with the SOLD system, photophysical parameters *k*_6_ (1.14 × 10^−5^ s^−1^) and *k*_7_ (235 μM^−1^·s^−1^) were found for in vitro solutions with NaN_3_. A linear relationship between SOLD singlet oxygen photon counts at 1270 nm and SOED-calculated reacted singlet oxygen (Equation (10)) was established for Photofrin for 523 nm light excitation. Based on our SOED calculations, a formula (Equations (11)–(13)) for converting cumulative SOLD signal measured at 523 nm to the corresponding [^1^*O*_2_]_rx_ at 630 nm was established using the optical properties at the two wavelengths in an ongoing MPM clinical protocol.

These results indicate that, with suitable correction for the tissue optical properties at the two wavelengths, there is excellent correlation between the direct (SOLD) and indirect (SOED) estimates of the singlet oxygen generated during PDT. Since, at the present time, the SOED approach is technically simpler and the instrumentation is significantly less expensive, this validation supports its use in clinical dosimetry. It should be noted, however, that the SOLD technique is intrinsically more robust in that no simplifying assumptions are required as in SOED. Hence, care must be taken in applying SOED to ensure that the treatment parameters lie within the range of validity of these assumptions. Furthermore, the validation of SOED must be carried out for each photosensitizer and set of clinical conditions. In the future, with developments such as our recent report of fiberoptic-coupled SOLD techniques based on novel superconducting nanowire single-photon detector technologies of direct SOLD, which currently can be considered as the laboratory Gold Standard for PDT dosimetry, may accelerate movement towards clinical utilization of SOLD alongside SOED. For type I PDT with photosensitizers, where a reactive oxygen species (e.g., oxygen radicals) other than singlet oxygen is the main cytotoxic agent, it is still possible to model the photophysical process using SOED, as described in a recent review [19]. However, it remains a challenge to find the value of photophysical parameters needed to describe the type I interactions for those photosensitizers.

## Figures and Tables

**Table 1:** Summary of photophysical parameters for Photofrin in vitro and in vivo.

Parameter	Definition	In Vitro	In Vivo
*ε* (cm^−1^·μM^−1^)	Photosensitizer extinction coefficient	0.0035 at 632 nm ^(1)^ 0.0089 at 523 nm ^(1)^	
*β* (μM)	Oxygen-quenching threshold concentration $\frac{k_{4}}{k_{2}}$ *	11.9 [34]	
*δ* (μM)	Low-concentration correction	25 ± 4.3 ^(2)^	33 [15]
*ξ* (cm^2^·mW^−1^·s^−1^)	Specific oxygen consumption rate $\xi =\Phi_{\Delta }\frac{\varepsilon }{h\nu }$	10.3 × 10^−3 (3)^ at 632 nm 24.8 × 10^−3 (4)^ at 523 nm	3.7 × 10^−3^ [34] at 632 nm 8.99 × 10^−3 (5)^ at 523 nm
*σ* (μM^−1^)	Specific photobleaching ratio where *σ = k*_1_*τ*_Δ_ *	(6.6 ± 7) × 10^−5 (2)^	7.6 × 10^−5^ [8,34]
*τ*_Δ_ (s)	Singlet oxygen lifetime $\frac{1}{k_{6}+k_{7}[A]}$ *	(9.4 ± 0.2) × 10^−6 (6)^	1.6 × 10^−7^ [35]
*τ_t_* (s)	Triplet state lifetime 1k4+k2[O23] *	(0.43 ± 0.03) × 10^−6 (7)^	1.5 × 10^−6 (7)^
*Φ*_Δ_	Singlet oxygen quantum yield	0.56 [36,37]	0.20 [19]

^(1)^ Measured from absorption spectroscopy; ^(2)^ Obtained from fitting shown in Figure 3c using Equation (7); ^(3)^ Calculated using *Φ*_Δ_ = 0.56 in water, *ε* = 0.0035 cm^−1^·μM^−1^, and *hν* = 3.2 × 10^−16^ mWs at 632 nm; ^(4)^ Calculated using *Φ*_Δ_ = 0.64 in MeOH, *ε* = 0.0089 cm^−1^·μM^−1^, and *hν* = 3.8 × 10^−16^ mWs at 523 nm; ^(5)^ Scaled in vivo value at 632 nm by *ε*(at 523 nm)/*ε*(at 632 nm); ^(6)^ Measured values from SOLD experiment when [*A*] = 0 (i.e., without NaN_3_ in MeOH solution); ^(7)^ Calculated from measured data using [^3^*O*_2_] = 40 μM for in vivo conditions and [^3^*O*_2_] = 169 μM for in vitro conditions. Thereby, *τ_t_* in vivo was estimated by determining *k*_2_ in vitro (1.3 × 10^4^ μM^−1^·s^−1^) and using *β* = *k*_4_/*k*_2_ = 11.9 μM and *1*/*τ_t_ = k*_2_(*β +* [^3^*O*_2_]); * The definition of the photophysical parameters are [19]: *k*_1_ = rate of photosensitizer (PS) photobleaching; *k*_2_ = rate of triplet PS quenching by ^3^O_2_; *k*_4_ = decay rate of triplet PS without ^3^O_2_; *k*_6_ = rate of ^1^O_2_ phosphorescence decay; *k*_7_ = rate of ^1^O_2_ quenching by substrate.

## Abbreviations

The following abbreviations are used in this manuscript:

PS	Photosensitizer
SOED	Singlet Oxygen Explicit Dosimetry
SOLD	Singlet Oxygen Luminescence Dosimetry
